# W. J. Smyth, M.D.S., L.D.S.

**Published:** 1903-04

**Authors:** H. J. Janlusz

**Affiliations:** 76 Fifth Avenue, New York City


### Obituary.

### W. J. SMYTH, M.D.S., L.D.S.

The New York papers stated that W. J. Smyth, M.D.S., L.D.S., committed suicide, Thursday, January 29. Having been associated with Dr. Smyth for a number of years, I have known him to be a good Christian. Dr. Smyth died of heart failure.

I wish to express my thanks to S. S. White, the Dentists' Supply Company, Ritter, Horace Jones, dental manufacturers, for their kind financial aid in assisting me to bury Dr. W. J. Smyth. He had no other friends or relatives in this country.

I hope you will find space for this, in justice to the late Dr. Smyth.

H. J. Janlusz.

76 Fifth Avenue, New York City.

